# Supplementary material for: Thermal sensitivity of field metabolic rate predicts differential futures for bluefin tuna juveniles across the Atlantic Ocean
Source: Nat Commun. 2023 Nov 27;14:7379. doi: 10.1038/s41467-023-41930-2 (PMC10682405; doi:10.1038/s41467-023-41930-2)
Supplement: Supplementary file 1 — Supplementary Information [file 41467_2023_41930_MOESM1_ESM.pdf]

Supplementary text

ABFT ecology / management background

Since the mid 1970s Atlantic BFT have been considered as two stocks separated by the 45°W management boundary, the western stock spawning in or near the Gulf of Mexico and potentially the adjacent shelf sea of the eastern US and the larger eastern stock spawning in or near the Mediterranean. The weight of evidence from conventional tagging, biologging, genetic and otolith microchemistry approaches supports this broad assessment although with uncertainty concerning the possibility of additional spawning areas (e.g. Slope Sea[1–3]) the degree and timing of trans-Atlantic migrations and the existence of sub-population structure within the Mediterranean[4]. Consequently, the international Commission for the Conservation of Atlantic Tunas (ICCAT) manages ABFT as two distinct stocks with natal fidelity, but allowing for intermixing of stocks. The importance of climate change impacts from a tuna fishery management perspective has recently been recognised by ICCAT with adoption of the first resolution on climate change.

Eastern and western populations of ABFT show different life history and growth trajectories with western populations typically slower growing. In both populations, spawning occurs in waters warmer than 21 °C[4], and larval tuna are associated with water temperatures from 20-28 °C. Consequently, spawning occurs earlier in the warmer western North Atlantic / Gulf of Mexico (April-June) than in the Mediterranean (May to July, east-west) [5,6]. Eastern ABFT remain in the Mediterranean sea for around a year, typically arriving in fisheries at the Bay of Biscay at a mean size of 60cm. The location and distribution of juvenile western ABFT is less well known[7], but larval and juvenile tuna in Gulf of Mexico are seldom found where SST exceeds 29°C [8]. Eastern ABFT mature earlier and at smaller body size, with an estimated 50% maturity at 3-8 years and at 130-200 cm, compared to uncertain but estimated > 8 years to reach maturity for western-origin ABFT. Differences in size at maturity between the two populations have been reported; in the western population, maturity is assumed to be achieved in fish no younger than nine years old or 185 cm curved fork length[9], while in the Mediterranean Sea 100% of ABFT show to be mature at >135 cm fork length (i.e., age 4-5 years)[10]. However, the similarity in growth rates of both components has raised doubts regarding the difference in age of sexual maturity, and novel approaches for assessing sexual

maturity in ABFT suggest that maturity ogives for ABFT originated from the western and eastern populations are indeed similar.

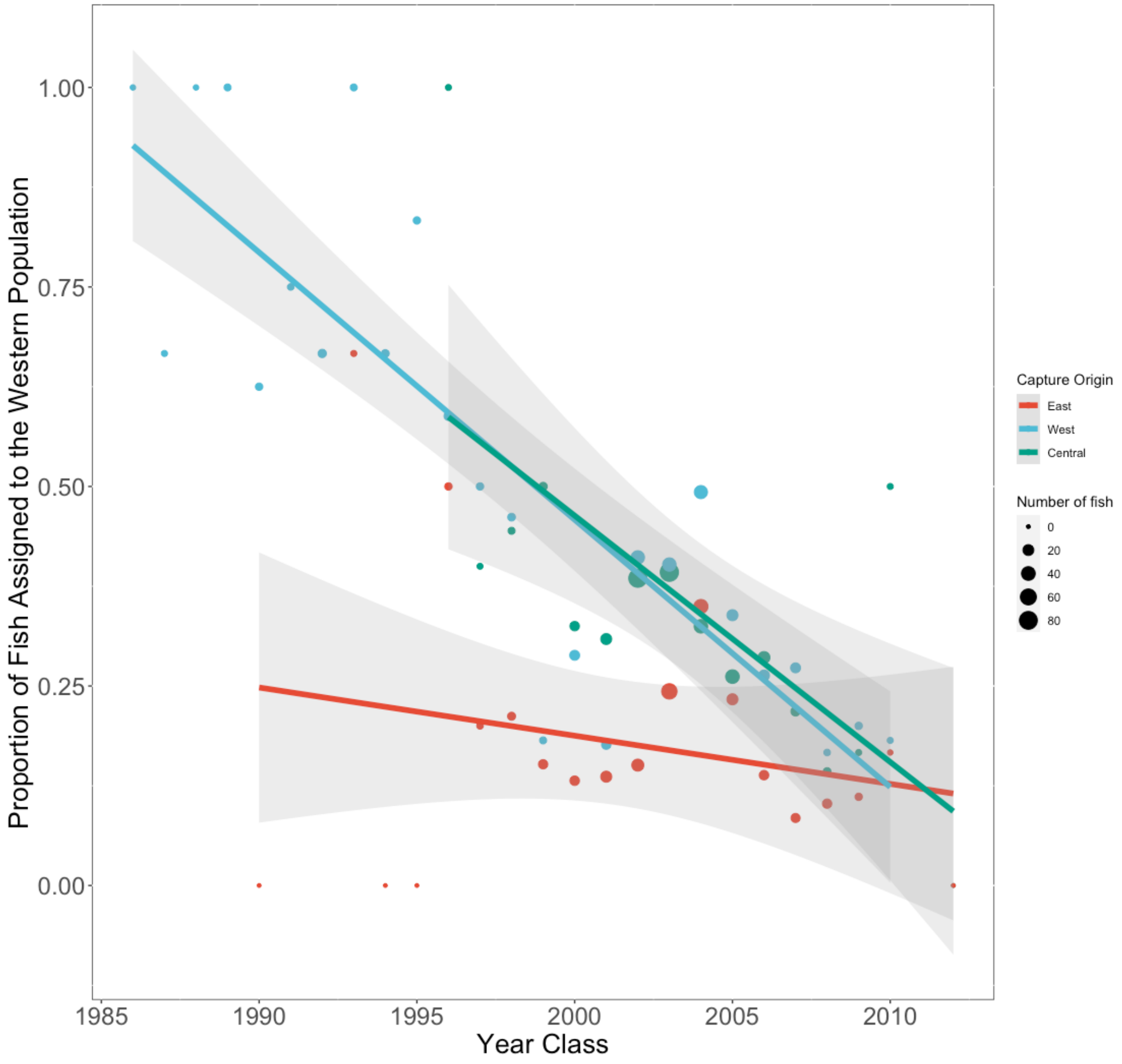

Supp. Fig. S1: Sustained reduction in the proportion of ABFT assigned to a western (Gulf of Mexico) origin seen in adult tuna captured in the East, West and Central regions of the North Atlantic. Year class represents birth year of the fish determined by otolith ageing. Data and code are available

Otolith FMR proxy background

Oxygen in otolith aragonite is deposited at, or close to, isotopic equilibrium with the ambient water, with a temperature-dependent fractionation such that the temperature of otolith precipitation can be estimated from knowledge of the isotopic compositions of the ambient water and the otolith. Carbon in otolith aragonite is not in isotopic equilibrium with the surrounding dissolved inorganic carbon[11–13]. Rather, carbon in the blood is a mixture of carbon derived from dissolved inorganic carbonate and carbon released from respiration of food. The stable isotope composition of these sources is very different: seawater carbon ($\delta^{13}C_{sw}$) values typically range between c.1 and -7‰ globally, while respiratory carbon ($\delta^{13}C_{resp}$) values generally vary between c. -10 to -25‰ in marine fishes. Otolith carbonate biomineral is formed from a mixture of these sources of HCO_3^- ions transported from blood into the biomineralising medium (endolymph fluid within the inner ear sacculus). The isotopic composition of inorganic carbon in blood, the endolymph fluid and otolith aragonite mineral is a weighted average of the relative contribution of respiratory carbon and seawater dissolved carbon[12,14–17]. Critically, as the rate of respiration of food sources increases, the relative proportion of respiratory carbon in blood increases. The proportion of respiratory carbon in otolith aragonite (otolith C_{resp}) can be determined from isotopic mass balance given estimates of the isotopic composition of diet and seawater carbon sources, providing a proxy measure of FMR averaged over the timeframe of otolith growth[18–25].

Climate projections.

Model	Nominal resolution	Institute
**ACCESS-CM2**	250 km	CSIRO (Commonwealth Scientific and Industrial Research Organisation, Australia), ARCCSS (Australian Research Council Centre of Excellence for Climate System Science)
**BCC-CSM2-MR**	100 km	Beijing Climate Center, Beijing, China
**CAMS-CSM1-0**	100 km	Chinese Academy of Meteorological Sciences, Beijing , China
**CESM2-WACCM**	100 km	National Center for Atmospheric Research, Climate and Global Dynamics Laboratory, Boulder, USA

**CESM2**	100 km	National Center for Atmospheric Research, Climate and Global Dynamics Laboratory, Boulder, USA
**CMCC-CM2-SR5**	100 km	Fondazione Centro Euro-Mediterraneo sui Cambiamenti Climatici, Italy
**CMCC-ESM2**	100 km	
**CNRM-CM6-1-HR**	25 km	CNRM (Centre National de Recherches Meteorologiques, Toulouse, France), CERFACS (Centre Europeen de Recherche et de Formation Avancee en Calcul Scientifique, Toulouse, France)
**CNRM-CM6-1**	100 km	
**CNRM-ESM2-1**	100 km	
**CanESM5-CanOE**	100 km	Canadian Centre for Climate Modelling and Analysis, Environment and Climate Change Canada, Victoria, Canada
**EC-Earth3-Veg-LR**	100 km	AEMET, Spain; BSC, Spain; CNR-ISAC, Italy; DMI, Denmark; ENEA, Italy; FMI, Finland; Geomar, Germany; ICHEC, Ireland; ICTP, Italy; IDL, Portugal; IMAU, The Netherlands; IPMA, Portugal; KIT, Karlsruhe, Germany; KNMI, The Netherlands; Lund University, Sweden; Met Eireann, Ireland; NLeSC, The Netherlands; NTNU, Norway; Oxford University, UK; surfSARA, The Netherlands; SMHI, Sweden; Stockholm University, Sweden; Unite ASTR, Belgium; University College Dublin, Ireland; University of Bergen, Norway; University of Copenhagen, Denmark; University of Helsinki, Finland; University of Santiago de Compostela, Spain; Uppsala University, Sweden; Utrecht University, The Netherlands; Vrije Universiteit Amsterdam, the Netherlands; Wageningen University, The Netherlands.
**EC-Earth3**	100 km	
**FGOALS-f3-L**	100 km	Chinese Academy of Sciences, Beijing, China
**FGOALS-g3**	100 km	
**GFDL-ESM4**	50 km	National Oceanic and Atmospheric Administration, Geophysical Fluid Dynamics Laboratory, Princeton, USA
**IPSL-CM6A-LR**	100 km	Institut Pierre Simon Laplace, Paris, France
**MIROC6**	100 km	JAMSTEC (Japan Agency for Marine-Earth Science and Technology, Kanagawa, Japan, AORI (Atmosphere and Ocean Research Institute, The University of Tokyo, Chiba, Japan), NIES

		(National Institute for Environmental Studies, Ibaraki, Japan), and R-CCS (RIKEN Center for Computational Science, Hyogo, Japan)
**MPI-ESM1-2-HR**	50 km	Max Planck Institute for Meteorology, Hamburg, Germany
**MPI-ESM1-2-LR**	250 km	
**MRI-ESM2-0**	100 km	Meteorological Research Institute, Tsukuba, Ibaraki, Japan
**TaiESM1**	100 km	Research Center for Environmental Changes, Academia Sinica, Nankang, Taipei, Taiwan
**UKESM1-0-LL**	100 km	Met Office Hadley Centre, Fitzroy Road, Exeter, Devon, UK

Table S1. List of models and corresponding institutions.

**Scenarios**	**Description**
**ssp126**	The sustainable and green pathway. Additional radiative forcing 2.6 W/m² by the year 2100. Update to the optimistic scenario RCP2.6 in CMIP5, with socio economic considerations
**ssp245**	Middle road. With an additional radiative forcing of 4.5 W/m² by the year 2100. An update to scenario RCP4.5 in CMIP5
**ssp370**	Additional radiative forcing of 7 W/m² by the year 2100, This scenario falls in the upper-middle part of the full range of scenarios.
**ssp585**	Additional radiative forcing of 8.5 W/m² by the year 2100, update of the CMIP5 scenario RCP8.5. higher end of future pathways, representing fossil-fueled development

Table S2. A Brief description of different future climate scenarios of the CMIP6 projections used in this study.

Limitations of data

The data analysed in this study were obtained opportunistically with respect to investigating thermal sensitivity of physiological performance data. Consequently the data are unbalanced sampling across years, ages and sites of origin. Samples of fish collected as fish older than 2 years of age are influenced by unbalanced collection across years of sampling and assigned juvenile origin. Most fish are assigned to a Mediterranean origin (Supp Fig S1), and consequently few fish experienced temperatures likely to result in thermal limitation. Additionally fish experiencing sub-optimal temps in the first year of life may be less likely to survive to adulthood. Consequently there are relatively few individuals available to constrain

thermal performance at temperatures in excess of 28°C. Furthermore, the gulf of Mexico and Mediterranean have different $\delta^{18}O_{water}$ values, but assigned origin for fish caught as adults in central Atlantic is based on otolith $\delta^{18}O$ values, creating a false break in inferred temperatures (Fig. 3D). These constraints do not apply to young of the year or yearling data which were sampled specifically in a single year and from known origin individuals to validate juvenile $\delta^{18}O$ values. Consequently, yearling data provide best estimates of the thermal sensitivity of C_{resp} values.

Supplementary References

1. Richardson, D. E. *et al.* Discovery of a spawning ground reveals diverse migration strategies in Atlantic bluefin tuna (Thunnus thynnus). *Proc Natl Acad Sci U S A* **113**, 3299–304 (2016).
2. Safina, C. Data do not support new claims about bluefin tuna spawning or abundance. *Proc Natl Acad Sci U S A* **113**, E4261–E4261 (2016).
3. Walter, J. F., Porch, C. E., Lauretta, M. V., Cass-Calay, S. L. & Brown, C. A. Implications of alternative spawning for bluefin tuna remain unclear. *Proc Natl Acad Sci U S A* **113**, E4259-4260 (2016).
4. Block, B. A. *The Future of Bluefin Tunas: Ecology, Fisheries Management, and Conservation*. (JHU Press, 2019).
5. Muhling, B. A. *et al.* Comparison between environmental characteristics of larval bluefin tuna Thunnus thynnus habitat in the Gulf of Mexico and western Mediterranean Sea. *Marine Ecology Progress Series* **486**, 257–276 (2013).
6. Gordoa, A., Fraile, I., Arrizabalaga, H. & Raventós, N. Growth of Mediterranean young-of-the-year bluefin tuna Thunnus thynnus (Scombridae): regional differences and hatching periods. *Scientia Marina* **85**, 61–69 (2021).
7. Galuardi, B. & Lutcavage, M. Dispersal Routes and Habitat Utilization of Juvenile Atlantic Bluefin Tuna, Thunnus thynnus, Tracked with Mini PSAT and Archival Tags. *PLOS ONE* **7**, e37829 (2012).
8. Muhling, B. A., Lee, S.-K., Lamkin, J. T. & Liu, Y. Predicting the effects of climate change on bluefin tuna (Thunnus thynnus) spawning habitat in the Gulf of Mexico. *ICES Journal of Marine Science* **68**, 1051–1062 (2011).
9. Restrepo, V.R. *et al.* 2010 Updated estimate of the growth curve of Western Atlantic bluefin tuna Aquat. Living Resour. 23: 335-342.
10. Corriero, A. *et al.* 2005 Size and age at sexual maturity of female bluefin tuna (Thunnus thynnus L. 1758) from the Mediterranean Sea. Journal of Applied Icthyology 21: 483-486.
10. Gauldie, R. W. Biological factors controlling the carbon isotope record in fish otoliths: Principles and evidence. *Comparative Biochemistry and Physiology Part B: Biochemistry and Molecular Biology* **115**, 201–208 (1996).
11. Kalish, J. M. 13cand 1 8 0 isotopic disequilibria in fish otoliths: metabolic and kinetic effects. 13.
12. Solomon, C. T. *et al.* Experimental determination of the sources of otolith carbon and associated isotopic fractionation. *Canadian Journal of Fisheries and Aquatic Sciences* **63**, 79–89 (2006).
14. Tohse, H. & Mugiya, Y. Sources of otolith carbonate: experimental determination of carbon incorporation rates from water and metabolic CO2, and their diel variations. *Aquatic Biology* **1**, 259–268 (2008).
15. Schwarcz, H. P. *et al.* Stable carbon isotope variations in otoliths of Atlantic cod (Gadus morhua). **55**, (1998).
16. Weidman, C. R. & Millner, R. High-resolution stable isotope records from North Atlantic cod. *Fisheries Research* **46**, 327–342 (2000).
17. McConnaughey, T. A., Burdett, J. ,. Whelan, J. F. ,. Paull, C. K. Carbon isotopes in biological carbonates: Respiration and photosynthesis. *Geochimica et Cosmochimica Acta* **61**, 611–622 (1997).
18. Chung, M.-T., Trueman, C. N., Godiksen, J. A., Holmstrup, M. E. & Grønkjær, P. Field metabolic rates of teleost fishes are recorded in otolith carbonate. *Commun Biol* **2**, 24 (2019).

19. Martino, J. C., Doubleday, Z. A., Chung, M.-T. & Gillanders, B. M. Experimental support towards a metabolic proxy in fish using otolith carbon isotopes. *J Exp Biol* **223**, jeb217091 (2020).
20. Trueman, C. N., Rickaby, R. E. M. & Shephard, S. Thermal, trophic and metabolic life histories of inaccessible fishes revealed from stable-isotope analyses: a case study using orange roughy *Hoplostethus atlanticus*: *hoplostethus atlanticus* life history. *J Fish Biol* **83**, 1613–1636 (2013).
21. Chung, M.-T., Trueman, C. N., Godiksen, J. A. & Grønkjær, P. Otolith δ13C values as a metabolic proxy: approaches and mechanical underpinnings. *Marine and Freshwater Research* **70**, 1747 (2019).
22. Chung, M.-T. *et al.* First measurements of field metabolic rate in wild juvenile fishes show strong thermal sensitivity but variations between sympatric ecotypes. *Oikos* **130**, 287–299 (2021).
23. Alewijnse, S. R. *et al.* Otolith-derived field metabolic rates of myctophids (Family Myctophidae) from the Scotia Sea (Southern Ocean). *Marine Ecology Progress Series* **675**, 113–131 (2021).
24. Sakamoto, T. *et al.* Contrasting life-history responses to climate variability in eastern and western North Pacific sardine populations. *Nat Commun* **13**, 5298 (2022).
25. Trueman, C. N., Chung, M.-T. & Shores, D. Ecogeochemistry potential in deep time biodiversity illustrated using a modern deep-water case study. *Phil. Trans. R. Soc. B* **371**, 20150223 (2016).
26. Trueman CN, Artetxe-Arrate I, Kerr LA, Meijers A, Rooker JR, Sivankutty R, Arrizabalaga, H, Belmonte A, Deguara S, Goñi N, Rodriguez-Marin E, Dettman DL, Neves Santos M, Saadet Karakulak F, Fausto Tinti F, Tsukahara Y, Fraile I. Thermal sensitivity of field metabolic rate predicts differential futures for bluefin tuna juveniles across the Atlantic Ocean. Zenodo. DOI:10.5281/zenodo.8305910. 2023.
